# Effects of anti-sclerostin antibody and running on bone remodeling and strength

**DOI:** 10.1016/j.bonr.2015.03.002

**Published:** 2015-04-11

**Authors:** H. Toumi, D. Benaitreau, S. Pallu, M. Mazor, R. Hambli, M. Ominsky, E. Lespessailles

**Affiliations:** a Service de Rhumatologie, Centre hospitalier régional d'Orléans, 1 rue Porte Madeleine, 45032 Orléans, France; b University of Orléans, I3MTO Laboratory, EA 4708, Hospital of Orleans, 1 rue Porte Madeleine, F-45032 Orléans, France; c Metabolic Disorders, Amgen Inc., One Amgen Center Dr., Thousand Oaks, 91320 CA, USA; d Prisme Institute, MMH, 8, Rue Leonard de Vinci, 45072 Orleans Cedex 2, France

**Keywords:** Anti-sclerostin antibody, Osteocyte, Osteoporosis, Physical exercise

## Abstract

Sclerostin antibody (Scl-Ab) represents a promising therapeutic approach to treat patients with osteoporosis. Purpose: The aim of this study was to investigate the effects of Scl-Ab, running and a combination of both on bone formation. Methods: Sixty female Wistar rats, aged 8 months were randomly assigned to five groups (subcutaneous injections performed twice a week): (1) (Sham): sedentary rats + saline, (2) (OVX): ovariectomized rats + saline, (3) (OVX + E): OVX rats + saline + treadmill training (5 times/week, 1 h/day), (4) (OVX + E + S): OVX rats + treadmill training + 5 mg/kg Scl-Ab and (5) (OVX + S): OVX rats + 5 mg/kg Scl-Ab. After 14 weeks, body composition, whole body and femoral BMDs were determined by DXA and serum was collected for analysis of osteocalcin and NTX. Bone microarchitecture was analyzed using μCT and bone strength was assessed at the femur mid-shaft in 3-point bending. Results: Running exercise decreased fat mass as well as the bone resorption marker NTX relative to the non-exercised control groups, effects that were associated with a prevention of the deleterious effects of OVX on whole body and femoral BMDs. Scl-Ab increased the bone formation marker osteocalcin, which resulted in robust increases in BMD and femoral metaphyseal bone volume to levels greater than in the Sham group. OVX + S + E group did not further impact on bone mass relative to the OVX + S group. At the cortical femur diaphysis, Scl-Ab prevented the decreases in bone strength after OVX, while exercise did not affect cortical strength. Conclusion: We suggest that while running on a treadmill can prevent some bone loss through a modest antiresorptive effect, it did not contribute to the robust bone-forming effects of Scl-Ab when combined in an estrogen ablation model.

## Highlights

• Weekly treatment with a low dose of Scl-Ab increased bone formation, bone mass, and bone strength in OVX Wistar mature rats.
• Running exercise decreased fat mass as well as the bone resorption marker NTX relative to the non-exercised control groups, effects that were associated with a prevention of the deleterious effects of OVX on whole body and femur BMDs.
• The combination therapy of Scl-Ab and running exercise did not further impact bone mass relative to the Scl-Ab, while it had moderate effect on bone physiology.

## 1 Introduction

The bone remodeling process constitutes a coupled activity of cells resorbing and forming bone (Moriishi et al., 2012). Disruptions in signaling pathways among these cells and alterations in their activity are considered to be part of the physiopathology of osteoporosis (Boyce et al., 2012, Cary et al., 2013). Several methods have been used to treat osteoporosis in order to reduce the risk of fractures (Boyce et al., 2012, Lippuner, 2012, Ng, 2009), including medications and increasing physical activity. Osteoporosis medications increase bone mass either by decreasing bone resorption (i.e. Bisphosphonates, Calcitonin, Selective Estrogen Receptor Modulators, e.g., Raloxifene, Estrogen/hormone therapy) (Migliaccio et al., 2007), by increasing bone formation (i.e. Teriparatide, a parathyroid hormone) (Ohtori et al., 2013), or by modulating the balance of both (Strontium ranelate) (Kaufman et al., 2013). Antibody-mediated inhibition of sclerostin, a pivotal negative regulator of bone formation (Li et al., 2009), represents a promising new therapeutic approach for the anabolic treatment of bone-related disorders, such as postmenopausal osteoporosis. Sclerostin is a protein produced primarily by osteocytes (Li et al., 2009, Bonewald, 2011), and inhibits osteoblastic activity on the surface of bone by binding to low-density lipoprotein receptors and inhibiting the Wnt/β-catenin signaling pathway (Li et al., 2009).

Regular exercise is a non-pharmacological option and considered an essential part of any osteoporosis treatment program (Iwamoto et al., 2004, Iwamoto et al., 2005, Hagihara et al., 2009, Honda et al., 2003). Bone formation and, consequently, Bone Mineral Density (BMD) are enhanced by physical activity in premenopausal women (Mosti et al., 2013, Anek et al., 2011). Physical activity increases the mechanical stresses on bone tissue (Cheung and Giangregorio, 2012, Niinimaki, 2012). Theoretically, the mechanical stress is detected by mechanoreceptors (i.e. integrins) (Batra et al., 2012, Xu et al., 2012) primarily on osteocytes, which ultimately transduce the mechanical signals into biological signals. Increased activity and stress can trigger bone modeling by directly increasing osteoblast activity, while a lack of stress can signal increased osteoclastic resorption. These processes are largely dependent on osteocyte activities, which control the communication towards and between osteoblast forming cells and osteoclast resorbing cells, perhaps in part through regulation of sclerostin expression (Poole et al., 2005, Li et al., 2008). Lin et al. (2009) reported that mechanical unloading of wildtype mice caused a decrease of Wnt/beta-catenin signaling activity accompanied by upregulation of Sost (Lin et al., 2009). However, the pathways by which mechanical forces are transduced to osteoclast and osteoblast activity are incompletely defined. Moreover, the amount and type of mechanical stress required remains debatable. It has been reported that running and jumping exercises produce changes in circulating levels of hormones such as growth hormone (GH) and insulin-like growth factor-1 (IGF-1), which have an anabolic effect on both bone and muscle (Iwamoto et al., 2004, Iwamoto et al., 2005, Hagihara et al., 2009, Honda et al., 2003). Among all types of exercise programs, high-impact exercise is thought to be greatly beneficial to bone (Iwamoto et al., 2004, Iwamoto et al., 2005, Hagihara et al., 2009, Honda et al., 2003). We hypothesized that a combination of a pharmacologic dose of Sclerostin Antibody (Scl-Ab) and running training might have a synergetic effect in osteoporosis treatment. The aim of the present study was to compare the effect of sclerostin antibody, running exercise and a combination of both on bone status in a female mature rat model.

## 2 Materials and methods

### 2.1 Animal treatment

The study protocol was approved by the Institutional Animal Care and Use Committee of our institution (agreement nos. C45-234-9 and 2011-11-2) and from the French National Institute of Health and Medical Research (INSERM) (approval ID: INSERM45-001). Sclerostin antibody was provided by Amgen (Thousand Oaks, CA, USA).

Sixty 8-month-old female Wistar rats (mean weight 341 ± 24 g) were purchased from Animal Production Janvier, Genest Saint-Isle, France. The animals were housed two per cage, in standard cages (30 × 28 × 20 cm^3^) and kept in a controlled environment (22 ± 2 °C, 12 h light–dark cycle) with free access to food and water. After one week of acclimation to the new environment, the rats were randomly ovariectomized or sham operated. After 2 months, the rats were randomly assigned to the 5 following groups (12 rats per group): (1) Sham: injected twice a week with saline and no exercise, (2) OVX: ovariectomized, injected twice a week with saline and no exercise, (3) OVX + E: ovariectomized, injected twice a week with saline and exercised treadmill running (see details below), (4) OVX + E + S: ovariectomized, injected with subcutaneous sclerostin antibody (5 mg/kg/day Scl-AbVI, twice a week) and exercised treadmill running and (5) OVX + S: ovariectomized, injected with sclerostin antibody (5 mg/kg/day, twice a week) and no exercise. The treadmill running protocol consisted of 1 h running, 5 days a week for a duration of 14 weeks. The maximum aerobic speed (MAS) of the rats was re-evaluated every 3 weeks. The MAS was determined as follows: after 10 min running at a low speed, the treadmill speed was gradually increased every 2 min until the rat refused or was no longer able to walk and start running. Training started with 10 min running at 50% of the MAS (from 13.43 to 24.15 m/min), followed by 5 cycles of 8 min at 80% of MAS (from 21.48 to 38.64 m/min) and 2 min at 50%. Exercise was performed 5 days a week for a total duration of 14 weeks. This protocol was chosen because a previous study showed it had a significant positive effect on bone mineral density in male Wistar rats (Boudenot et al., 2012). One rat from the OVX group died before the end of the experiment and was thus excluded.

After 14 weeks of treatment, animals were sacrificed by exsanguination under anesthesia with *pentobarbital*. The procedure for the care and killing of the animals was in accordance with the European Community standards on the care and use of laboratory animals. After sacrifice, femurs and tibias were removed and cleared of surrounding soft tissue; blood samples were centrifuged and the serum was kept at − 20 °C before ELISA analysis.

### 2.2 Body weight, fat mass and BMD measurements

Whole body weight, fat mass and BMD were evaluated by DXA for the whole body and for the right femur on a Discovery scanner (Hologic, Bedford, Massachusetts, USA). The parameters of body composition and BMD were determined using the scanner's APEX software.

### 2.3 Bone microarchitecture/macroarchitecture

**Fig. 1:**
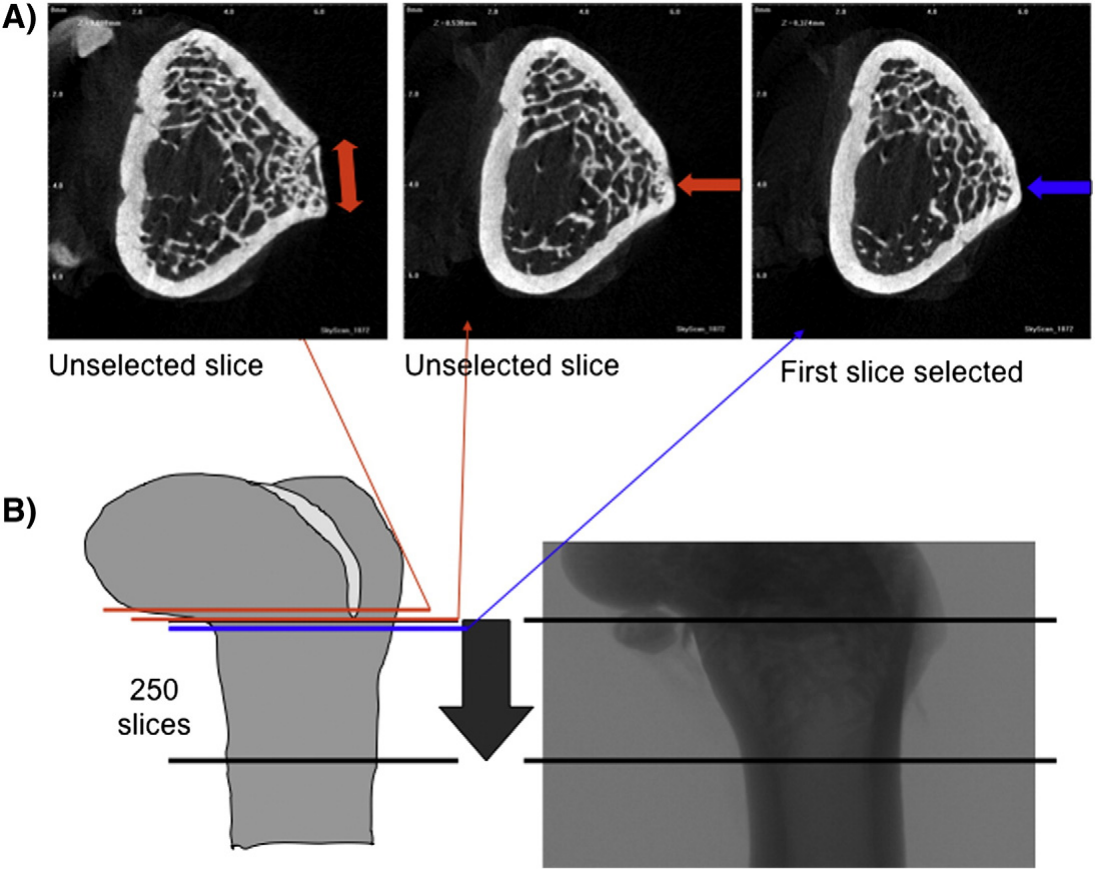
Radiographic projection of the distal femoral metaphysis acquired by microcomputed tomography. A: Transversal sections of the femoral metaphyseal area, left and center images: red arrow show a furrow in the cortical part to explain unselected slices; right image presents no furrow (blue arrow) and constitutes the first selected slice. B: Schematic representation of the region of interest that corresponds to 250 slices from the distal growth plate (upper black line) to the shaft proximally (lower black line).

Femoral microarchitecture was analyzed using a micro-computed tomograph (μCT, Skyscan 1072; Skyscan, Kontich, Belgium) and following the protocol described previously by Bonnet et al. (2006). The X-ray source was set at 85 kV and 100 μA, with a pixel size of 11.16 μm. Four hundred projections were acquired over an angular range of 180° (angular step of 0.45°). For each sample, 250 slices were selected from the distal metaphysis. Details on how the 250 slices of the distal metaphysis were consistently selected relative to anatomical locations are shown in Fig. 1. The trabecular bone region of interest (ROI) was extracted by drawing ellipsoid contour with CT analyzer software (Skyscan). The following parameters were measured: bone volume/tissue volume (BV/TV; expressed in percentage), trabecular spacing (Tb.Sp; μm), trabecular number (Tb.N; 1/mm) and trabecular thickness Tb.Th (mm). The distal femur cortex was analyzed using Matlab Software as described previously by Toumi et al. (2012), using a thresholding range developed previously (Toumi et al., 2012). Cortical thickness (Ct. thickness), cortical porosity (Ct. porosity), and cortical volume (Ct. volume) were calculated on the binary images based on two-dimensional (2D) analysis. The porosity was calculated as the ratio of the total area of pores to the total area of cortical bone space.

### 2.4 Bone biochemical markers

Bone turnover markers were analyzed in the terminal serum samples. Osteocalcin was analyzed as a marker of bone formation using a commercial Rat Osteocalcin EIA kit (IDS, France). Intra- and inter-assay CV were respectively 5.0% and 5.5% and the detection limit was 50 ng/ml. Telopeptide N of type I collagen (NTX) was analyzed as a marker of bone resorption. Serum samples were analyzed in duplicate using an ELISA Kit (NTX osteomark serum; TECO medical SARL, Versailles, France). Intra- and inter-assay CV were 4.6% and 6.9% respectively and the detection limit was 3.2 nM Bone Collagen Equivalents/L (nM BCE/L).

### 2.5 Bone mechanical testing

Mechanical properties of the femur were assessed by a three-point bending test. Each femur was secured on the two lower supports on the anvil of a Universal Testing Machine (Instron 3343; Instron, Melbourne, Australia). The distance between the two supports was 20 mm. Loading point contacted the midpoint of the femoral diaphysis in an antero-posterior direction at a speed of 1 mm/min. Load-displacement curves were collected using specialized Instron 3343 software. Ultimate load (the maximal force supported by bone before fracture, N) and stiffness (extrinsic rigidity; N/mm), were calculated according to the method described previously by Turner and Burr (Turner and Burr, 1993).

### 2.6 Statistics

Numerical variables were expressed as mean ± SEM. For each parameter, the group's normality was tested using a Shapiro Wilk test. The homogeneity of the variances was tested to compare groups using a Fisher F test. Parameters were classified to parametric and non-parametric. For the parametric values, a two-way analysis of variance (ANOVA) was used. The two factors in the ANOVA were sclerostin antibody treatment and exercise, while the dependent variables were the total body weight, the Ct. thickness and the NTX concentration at the end of the study. To test the level of significance, a PLSD Fisher post hoc test when significant. A Kruskal Wallis test was used for the nonparametric values and groups were subsequently compared using the Dunn's post hoc test. Values of p < 0.05 were considered statistically significant.

## 3 Results

**Fig. 2:**
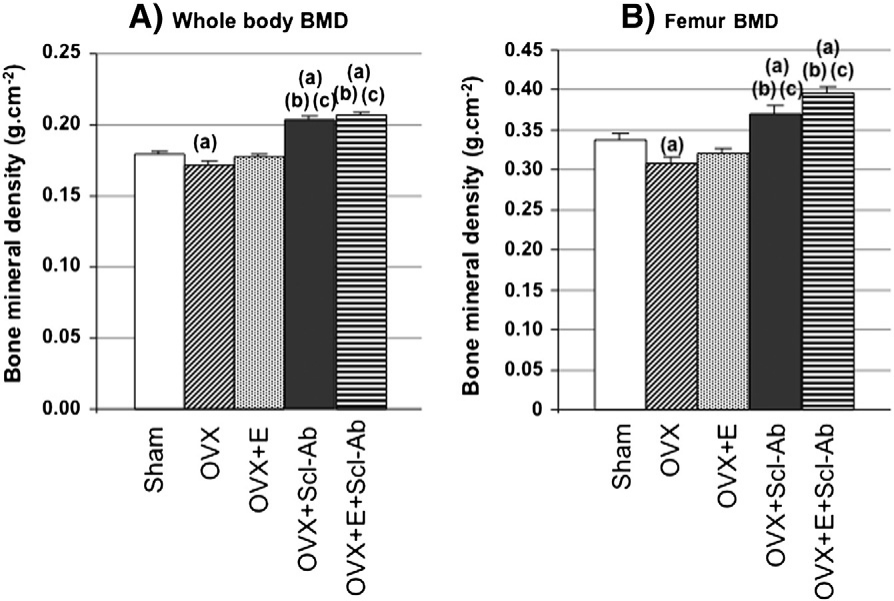
Whole body (A) and femoral (B) BMDs measured by DXA. All values represent mean ± SEM. The critical p-value (p) was 0.05; a, b, c represent significant differences vs. Sham, OVX and OVX + E respectively.

**Fig. 3:**
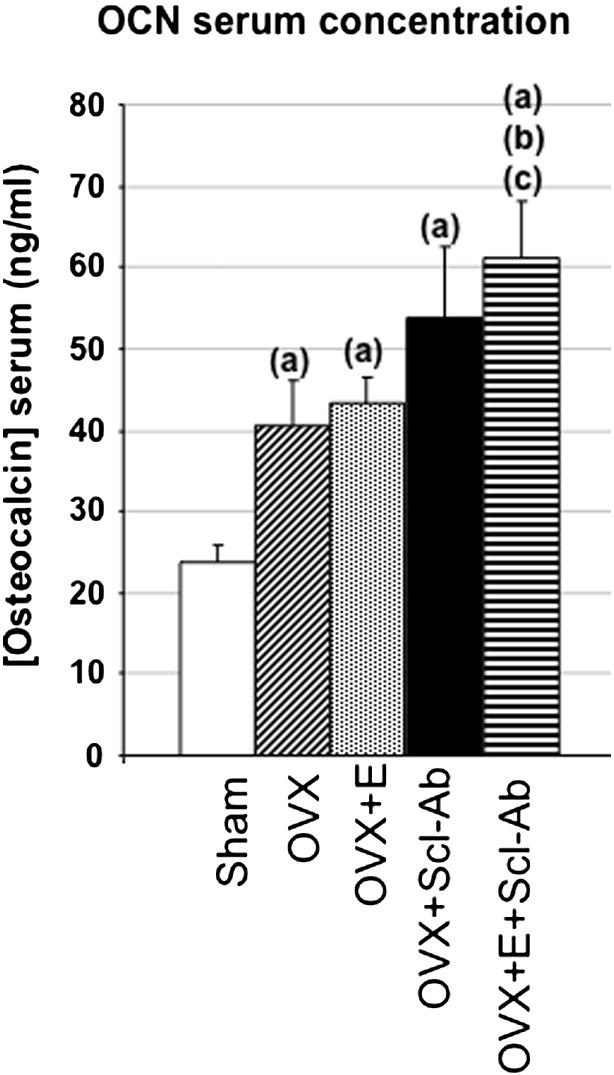
Serum osteocalcin (OCN) concentration. All values represent mean ± SEM. The critical p-value (p) was 0.05; a, b, c represent significant differences vs. Sham, OVX and OVX + E, respectively.

**Fig. 4:**
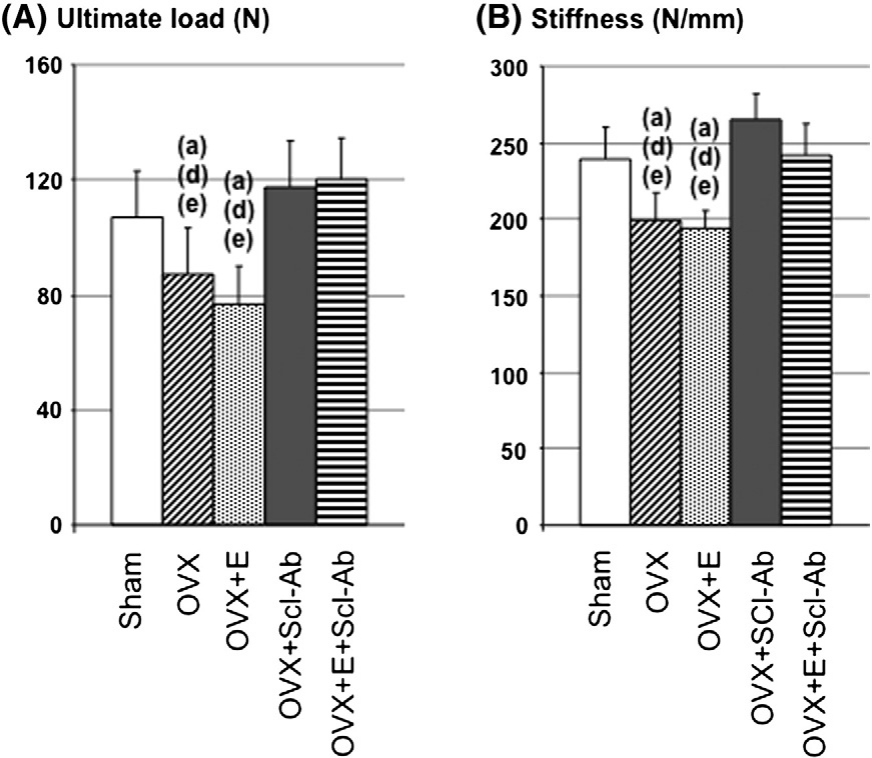
Bone mechanical parameters. Mechanical properties of the femur were assessed by a three-point bending test. A. Ultimate load (the maximal force supported by bone before fracture), B. Stiffness (extrinsic rigidity). All values represent mean ± SEM. The critical p-value (p) was 0.05; a, d, e represent significant differences vs. Sham, OVX + E + S and OVX + S respectively.

The overall results showed that exercise decreased fat mass as well as the bone resorption marker NTX relative to the non-exercised control groups, effects that were associated with a prevention of the deleterious effects of OVX on whole body and femur BMDs. Scl-Ab increased the bone formation marker osteocalcin, and resulted in robust increases in BMDs and femur metaphyseal bone volume to levels greater than in the Sham group. The addition of exercise in the OVX + S + E group did not further impact bone mass relative to the OVX + S group. At the cortical femur diaphysis, Scl-Ab prevented the decreases in bone strength after OVX, while exercise did not affect cortical strength. These results are summarized in Fig. 2, Fig. 3, Fig. 4, Table 1, Table 2, Table 3, Table 4 and detailed below.

### 3.1 Body weight and composition (Table 1, Table 2)

The body weight measured at the end of the study revealed that the OVX group showed a significant body weight gain compared with the sham group (p < 0.01). Overall body weight measured at the end of the study was lower in the OVX + E + S group compared with the OVX group (p < 0.01). Similar results were obtained for fat mass, with both trained OVX groups having a significantly lower fat mass compared to non-trained OVX. Finally, there was no significant difference in the lean mass between all the groups.

### 3.2 Bone mineral density (Fig. 2)

Both whole body and femoral BMDs were significantly lower in the OVX group compared to Sham controls. Exercise did not induce a significant improvement in BMD compared to OVX controls, but there was no significant difference between OVX + E and Sham. Scl-Ab resulted in significant increases in BMD compared to all other groups, though a significant additive effect of exercise was not observed relative to the OVX + S group.

### 3.3 Bone microarchitecture and macroarchitecture (Table 2, Table 3, Table 4)

As expected, trabecular bone volume in the distal femur metaphysis was decreased in the OVX group vs Sham controls (Table 3). Trabecular BV/TV and Tb.Th were significantly higher for both Scl-Ab groups compared to all other groups (Sham, OVX and OVX + E). Cortical volume and cortical thickness in the femur metaphysis were significantly higher in sclerostin antibody groups (OVX + S and OVX + E + S) compared to all groups (Sham, OVX and OVX + E), and neither exercise nor ovariectomy affected these parameters (Table 2, Table 3, Table 4). Cortical porosity was the highest in the OVX + E group compared to OVX and Sham controls, and Scl-Ab prevented the significant increase in porosity with training.

### 3.4 Bone biochemical markers (Table 2, Fig. 3)

OCN was significantly higher in all OVX groups compared to Sham, further increased with Scl-Ab, resulting in a significantly higher value in OVX + E + S compared to OVX and OVX + E. NTX was significantly lower in trained groups relative to OVX, while Scl-Ab alone resulted in a non-significant decrease. Moreover NTX was significantly lower in OVX + E + S than in OVX + S.

### 3.5 Bone mechanical testing (Fig. 4)

At the femur diaphysis, OVX resulted in significant decreases in ultimate load and stiffness relative to Sham controls, while sclerostin antibody significantly improved these parameters compared to OVX controls. Training alone had no significant effect on strength, and did not significantly add to the effect of Scl-Ab treatment.

## 4 Discussion

While running exercise is recommended for optimum cardiovascular and overall health and anything that gets your heart rate into your target heart rate zone will work, the efficacy of running exercise therapy on bone status and particularly in combination with osteoporosis therapeutics is relatively underreported. The current study examined the effect of sclerostin antibody, running exercise and a combination of both on bone mass in 10-month old OVX rats. Our findings confirm that running exercise decreased fat mass as well as the bone resorption marker NTX relative to the non-exercised control groups, effects that were associated with a prevention of the deleterious effects of OVX on whole body and femur BMDs. Scl-Ab increased the bone formation marker osteocalcin, and resulted in robust increases in areal BMD and femur metaphyseal bone volume to levels greater than in the Sham group. The combination of Scl-Ab and running did not further impact bone mass and strength relative to Scl-Ab alone, though NTX and fat mass were also reduced.

A lower dose than typically been used in animal models (5 mg/kg twice a week) of Scl-Ab was sufficient to increase cortical volume, BMD, BV/TV and Tb.Th. These findings are consistent with the effects of the typical Scl-Ab dose used in animal studies (25 mg/kg twice a week) which have been shown to significantly increase bone formation, bone volume, and bone strength in multiple rodent models (summarized in Ke et al. Endo Reviews) (Ke et al., 2012). The use of the 5 mg/kg dose is closer to the highest dose level (3 mg/kg) tested in Phase 2 clinical studies (McClung et al., 2014), and its more modest effect was considered optimal to allow potential additive effects with exercise (Tian et al., 2011, Agholme et al., 2011).

Treadmill exercise has primarily been used to evaluate the effects of running on bone homeostasis (Barengolts et al., 1993, Barengolts et al., 1994, Peng et al., 1994, Peng et al., 1997). Previous studies have demonstrated that treadmill running increased bone formation and decreased bone resorption in growing rats, resulting in increases in BMD and trabecular microarchitecture in weight bearing sites (Barengolts et al., 1994, Peng et al., 1994). In our female mature model, while BMD of OVX exercised rats increased to approximately the same extent as Sham, and NTX decreased, our running exercise had a more modest effect on BMD compared to previous results from jumping rat models (Iwamoto et al., 2004, Iwamoto et al., 2005, Hagihara et al., 2009, Honda et al., 2003). This difference may be related to the significantly higher principal tension, compression and shear strain and strain rates with jumping compared to treadmill running (Milgrom et al., 2000). It has been reported that high-impact exercise has greater beneficial effect on bone formation and strength (Honda et al., 2003), while running on a treadmill may not create stress levels high enough to reach the bone modeling threshold required, as described by Frost (Frost, 1997) in his mechanostat theory. In addition, it is possible that estrogen loss may have shifted the bone-modeling threshold in OVX rats, thus reducing the anabolic effect of treadmill running in the current study. However, previous studies have reported that rats with high bone turnover (i.e. estrogen-deficient) are more sensitive to loading compared with normal rats (Barengolts et al., 1993, Frost, 1997). Consistent with the current findings, running exercise resulted in reductions in urinary NTX levels in postmenopausal women with osteopenia, an effect that was correlated with improvements in lumbar BMD (Yamazaki et al., 2004).

In addition to the load-based effects of exercise on the regulation of bone homeostasis, it is possible that some of the preventive bone effects could be attributed to the inhibition of fat mass accumulation post-OVX. A recent publication has focused on the interactions between fat and bone (Reid, 2008). Although a direct role of visceral fat in regulating bone resorption has not been established, obesity has been linked to chronic inflammation that could result in upregulation of proinflammatory cytokines or hormones and thus increased osteoclastogenesis (Cao, 2011). However, weight loss has been reported to result in elevations in bone resorption markers in humans, an effect hypothesized to be associated with reduced skeletal loading rather than loss of fat. In any exercise model it is challenging to separate out the effects of loading from other effects on skeletal physiology. In fact, in the short term, exercise has been reported to increase bone resorption markers in humans (Welsh et al., 1997), while the opposite is true in the longer-term (Phoosuwan et al., 2009), suggesting that the effects of exercise on bone resorption may not be solely loading-based. Thus, the interactions between increasing fat mass and regulation of bone turnover require further exploration.

The expression of sclerostin in bone is also regulated by the mechanical loading environment (Robling et al., 2008). Thus, sclerostin is considered an important mediator of the anabolic effects of loading, and in the catabolic effects of unloading. Herein, the combination therapy of Scl-Ab and running exercise did not further impact bone formation and strength relative to the Scl-Ab, perhaps due to the robust bone effects of Scl-Ab relative to our treadmill exercise model.

In summary, weekly treatment with a low dose of Scl-Ab increased bone formation, bone mass, and bone strength in OVX Wistar mature rats. Running exercise decreased fat mass as well as the bone resorption marker NTX relative to the non-exercised control groups, effects that were associated with a prevention of the deleterious effects of OVX on whole body and femur BMDs. The combination therapy of Scl-Ab and running exercise did not further impact bone mass relative to the Scl-Ab, while it had moderate effect on bone physiology.

## Figures and Tables

**Table 1:** Fat mass and total body weight measured at the end of the study. All values represent mean ± SEM. (a) Significant difference compared to Sham group (p < 0.05). (b) Significant difference compared to OVX group (p < 0.05).

	Sham	OVX	OVX + E	OVX + S	OVX + E + S
Total body weight (g)	335 ± 6 (n = 12)	404 ± 12 (a) (n = 11)	371 ± 10 (a) (n = 12)	372 ± 12 (a) (n = 12)	358 ± 10 (b) (n = 12)
Fat mass (g)	57.95 ± 2.99	118.27 ± 11.19 (a)	88.63 ± 5.49 (a,b)	116.12 ± 24.94 (a)	72.19 ± 6.28 (b)

**Table 2:** Parametric data for the five groups: Cortical Thickness (Ct.Thickness), NTX serum level measured at the end of the study. (a) Significant difference compared Sham group (p < 0.05). (b) Significant difference compared OVX group (p < 0.05). (c) Significant difference compared OVX + E group (p < 0.05). (e) Significant difference compared OVX + S group (p < 0.05). All values represent mean ± SEM. P_interaction_: Statistical interaction between the effects of sclerostin antibody and exercise (< 0.05). P_Scl-Ab_: Statistical degree of the influence of sclerostin antibody (< 0.05). P_exercise_: statistical degree of the influence of exercise (< 0.05). There was no statistical interaction between the effects of sclerostin antibody and exercise for the three parameters.

Parametric data	Sham	OVX	OVX + E	OVX + S	OVX + E + S	P SclAb	P exercise	P interaction
Ct. Thickness (μm)	506.73 ± 12.84 (n = 12)	541.30 ± 14.33 (n = 11)	511.14 ± 12.39 (n = 11)	650.08 ± 17.55 (a,b,c) (n = 12)	665.84 ± 16.20 (a,b,c) (n = 12)	< 0.0001	0.6431	0.1442
NTX serum level (nM)	12.14 ± 0.69 (n = 12)	13.31 ± 0.62 (n = 11)	11.08 ± 0.92 (b) (n = 12)	11.97 ± 0.62 (n = 11)	9.18 ± 0.60 (a,b,e) (n = 12)	0.0281	0.0010	0.6926

**Table 3:** Trabecular microarchitectural parameters in distal femoral metaphysis measured by microcomputed tomography (μCT). All values represent mean ± SEM. Bone volume/tissue volume BV/TV (%), trabecular thickness Tb.Th (mm), trabecular number Tb.N (1/mm), trabecular spacing Tb.Sp (μm). The critical p-value (p) was 0.05; a, b, c represent significant differences vs Sham, OVX and OVX + E respectively.

	Sham	OVX	OVX+E	OVX+S	OVX+E+S
					
BV/TV (%)	15.60 ± 1.57	7.09 ± 1.13 (a)	8.13 ± 0.81 (a)	28.77 ± 3.82 (a,b,c)	26.07 ± 3.46 (a,b,c)
Tb.Th (mm)	0.1040 ± 1745	0.0930 ± 0.0023 (a)	0.0964 ± 0.0024 (a)	0.1675 ± 0.0066 (a,b,c)	0.1803 ± 0.0052 (a,b,c)
Tb.N (1/mm)	1.525 ± 0.1745	0.746 ± 0.1077 (a)	0.851 ± 0.0884 (a)	1.694 ± 0.2109 (b,c)	1.437 ± 0.1823 (b,c)
Tb.Sp (mm)	0.724 ± 0.0931	0.879 ± 0.0834	0.807 ± 0.073	0.787 ± 0.096	0.763 ± 0.064

**Table 4:** Microarchitectural at the distal femoral metaphysis measured by microcomputed tomography (μCT). All values represent mean ± SEM, the critical p-value (p) was 0.05. Cortical volume (Ct volume) and cortical porosity (Ct porosity); a, b, c represent significant differences vs. Sham, OVX and OVX + E respectively.

	Sham	OVX	OVX+E	OVX+S	OVX+E+S
					
Ct. Volume (μm^3^)	4978.7 ± 1062.38	50409.5 ± 1609.04	50852.9 ± 924.63	63237.6 ± 1533.37 (a,b,c)	66393.4 ± 1705.85 (a,b,c)
Ct. Porosity (%)	0.05367 ± 0.0009	0.0547 ± 0.00108	0.06269 ± 0.00253 (a,b)	0.05082 ± 0.00237 (c)	0.5015 ± 0.00119 (a,b,c)
